# If Numbers Can Speak, Who Listens? Creating Engagement and Learning for Effective Uptake of DRR Investment in Developing Countries

**DOI:** 10.1371/currents.dis.ab5922892b54a68f7315e967f6dd3406

**Published:** 2016-06-13

**Authors:** Junko Mochizuki, Adriana Keating, Reinhard Mechler, Callahan Egan, Stefan Hochrainer-Stigler

**Affiliations:** Department of Risk and Resilience, International Institute for Applied Systems Analysis, Laxenburg, Austria; Department of Risk and Resilience, International Institute for Applied Systems Analysis, Laxenburg, Austria; International Institute for Applied Systems Analysis, Laxenburg, Austria; Vienna University of Economics and Business, Vienna, Austria; Department of Risk and Resilience, International Institute for Applied Systems Analysis, Laxenburg, Austria; Department of Risk and Resilience, International Institute for Applied Systems Analysis, Laxenburg, Austria

## Abstract

Introduction: With a renewed emphasis on evidence-based risk sensitive investment promoted under the Sendai Framework for Disaster Risk Reduction 2015-2030, technical demands for analytical tools such as probabilistic cost-benefit analysis (CBA) will likely increase in the foreseeable future. This begs a number of pragmatic questions such as whether or not sophisticated quantitative appraisal tools are effective in raising policy awareness and what alternatives are available.

Method: This article briefly reviews current practices of analytical tools such as probabilistic cost-benefit analysis and identifies issues associated with its applications in small scale community based DRR interventions.

Results: The article illustrate that while best scientific knowledge should inform policy and practice in principle, it should not create an unrealistic expectation that the state-of-the art methods must be used in all cases, especially for small scale DRR interventions in developing countries, where data and resource limitations and uncertainty are high, and complex interaction and feedback may exist between DRR investment, community response and longer-term development outcome.

Discussion: Alternative and more participatory approaches for DRR appraisals are suggested which includes participatory serious games that are increasingly being used to raise awareness and identify pragmatic strategies for change that are needed to bring about successful uptake of DRR investment and implementation of DRR mainstreaming.

## Introduction

Amid the rising costs of natural disasters observed globally, emerging international consensus holds that a primary remedy to the lack of appropriate risk management – and ex-ante disaster risk reduction (DRR) investment in particular – can be found in better communication regarding the cost-effectiveness of DRR investment 1 ^,^ 2. Over the past two decades, natural disasters have affected a total of 4.4 billion people worldwide, causing as much as $2 trillion in economic losses 3. By 2030, accelerating urbanization, continued asset build-up in hazard-prone areas and other adverse consequences of poorly managed development are expected to double the economic losses from natural disasters globally^4^. To curtail these trends in risk creation, ex-ante quantification, evaluation and the integration of disaster risk into development are increasingly seen as crucial. ‘DRR investment saves’ has been a major slogan in such discourse, where the lack of awareness and scientific evidence are seen as the major obstacles hindering the rational decision to invest in ex-ante protection of our wellbeing, livelihoods and productive assets^5^.

The general premise that improved risk knowledge will lead naturally to enhanced DRR investment is not only theoretically debatable^6^ as has been critiqued by numerous behavioural and political economic studies which explain why we continue to underinvest in DRR *despite* the knowledge of its net benefits^7^^,^ 8 ; it has also created a practical issue in which increasingly sophisticated enquiries – often beyond the capacities of developing country practitioners – are called for to present scientific evidence regarding the costs and benefits of DRR investment 9 . While the concept and use of evidence-based assessments is advocated, the practical challenges of implementing such analyses in developing country contexts are, until today, insufficiently acknowledged.

A quantitative method known as probabilistic cost-benefit analysis (CBA) is often considered the state-of-the-art in the appraisal of DRR policies, in which probability distributions relating hazard return periods and loss estimates are calculated 10. The use of such CBAs has expanded considerably in the recent years where not only large scale public investment, but also smaller community-scale DRR interventions are increasingly evaluated using this methodology 11 ^,^ 12 ^,^ 13. Despite the considerable complexity, uncertainty, resource requirements, common measurements such as benefit-cost (B/C) ratio and Net Present Value (NPV) have routinely been used to justify, and raise awareness for, an increased investment in ex-ante DRR^13^ .

Calls for more sophisticated risk assessments, especially in light of climate change, however begs a number of pragmatic questions – such as whether or not sophisticated quantitative appraisal tools are effective in raising policy awareness and what alternatives are available. These questions are particularly germane in the context of developing countries where data and resource limitations, computational needs and uncertainty are high, and complex interaction and feedback may exist between DRR investment, community response and longer-term development outcome.

## When risk-based decision tools should be more than awareness-raising

‘DRR investment saves’ is hardly a new story: good technical evidence exists within developed countries 14, and more stock-taking are recurrently taking place in developing countries 12 ^,^ 13 . Moreover, what researchers have learned over the years is that the core issues behind continued under-investment in DRR are more than the lack of knowledge: not only cognitive issues, but behavioural gaps of failing to act, institutional barriers of not having appropriate mandates, and other political economic barriers all hinder DRR uptake. Therefore, using alternative and more sophisticated appraisal approaches *per se* is unlikely to help. While CBA has been frequently required by national and international bodies, available evidence suggests this has led to limited learning during project appraisal and implementation^15^ .

The true utility of appraisal methods such as CBA will likely be realized when it is used for tangible policy and investment decisions rather than mere awareness-raising. However, there are a number of practical challenges for such tangible application in developing countries. The lack of local capacities to collect and analyse hazard-, exposure- and vulnerability information is a major issue: whether downscaling efforts are made to climate hazard estimations or national average parameters are used to construct particular cost estimates, these will all contribute to increasing uncertainty bounds, and making proper sense of such uncertainty becomes a big challenge. Moreover, as these efforts require highly sophisticated analytical skills, which are often handled by external consultants, thereby removing the process of evaluation from community deliberation and learning. Methods such as CBA can be conducted in a participatory manner 16 , however, this often involves an elaborative process and learning potential may be limited. CBA is of course hardly immune from the usual caveats in the developing countries, including the lack of consideration for distributional consequences, limited applicability of shadow pricing, issues of high discount rates and costing of intangibles 13 .

## At what cost?

Given the complexity and uncertainty involved, it is unlikely that such a sophisticated approach will be appropriate for all cases, especially for small scale DRR project appraisal in developing countries. Missing reliable past disaster damage and loss estimates, probabilistic assessment of community-based interventions often rely on local knowledge: the establishment of baseline risk from annual to 2-5 year events may be feasible based on local residents’ recollection, however such methods quickly become infeasible for higher return period events such as 100 or 500 year events that are needed to accurately estimate the risk of extremes.

Furthermore, these issues are compounded when the appraisal is required to integrate the future impact of climate risk, which is particularly uncertain at the local scale. This requirement may be understandable when donor funding is extended under climate change adaptation. However, when such demands are made on tools such as probabilistic CBA, it adds a further layer of uncertainty on top of the existing hazard variability and local exposure and vulnerability dynamics which themselves are often poorly understood. Even in ideal circumstances, state-of-the art risk assessments are limited int their ability to offer robust future forecast, since the further they need to project into the future, the larger the uncertainty bounds become for hazards, exposure and vulnerability. Finding meaningful interpretations therefore becomes progressively difficult.

## When not all evidence has to be numbered

In light of these practical constraints, communities may instead use more locally appropriate methodologies to raise awareness and to plan for improved DRR practice in the context of climate change. Practitioners may raise local awareness regarding the effectiveness of 'soft' DRR measures vs. ‘hard’ DRR measures, by simply comparing the direct damages avoided such as crop loss, human injury and the deaths avoided based on the knowledge of past disaster events.

Furthermore, interactive methods such as participatory ‘serious’ gaming may be used to address existing barriers and uncertainty. Instead of reducing DRR benefits and costs into a single metric such as B/C ratio and NPV, this type of participatory analysis takes the advantage of its complexity and uncertainty – by turning them into a fun and creative virtual space where community members and practitioners together explore potential solutions for DRR. These system-thinking based tools can be used to identify barriers to action such as cognitive and behavioral stumbling blocks (e.g. resistance to changes and deviation from existing practices and social norms) or institutional and political barriers that may challenge the implementation of DRR^17^^,^ 19 ^,^ 21 ^,^ 22 ^,^ 23 . These types of approaches are particularly effective at challenging the many preconceived notions that are at the heart of policy inaction: as a recent study of game-based study in Mozambique, Uganda and Ethiopia show those who saw nothing but obstacles to change prior to gaming-interventions were able to identify and implement pragmatic changes to their community adaptation planning such as involving wider sectors in discussions and designing DRM plans based on potential, as opposed to past, hazard occurrence.^21^ Prior understanding of institutional and social contexts are crucial in designing and implementation and communications and reflections during and after gaming-sessions are one of the most important element in bringing about attitude, behavioral and institutional changes.^21^^,^ 23 The training needs for skilled facilitators and time commitment of community consultations are of course high, but another strength is that these types of analyses may be built upon existing monitoring and evaluation techniques used by community-based practitioners such as vulnerability and capacity assessment (VCA)^11^^,^ 18 .

## Awareness-raising and appraisal tools—getting our expectations right for Post-2015 DRR agendas

Looking beyond 2015, the demand for risk-based decision-making will likely increase. While best scientific knowledge should inform policy and practice in principle, it should not create an unrealistic expectation that the state-of-the art methods must be used in all cases. If decision-making tools, such as probabilistic CBAs, are to be useful (rather than be constraining), there are number of areas where further research and capacity building are required. First and foremost, the use of quantitative risk appraisal tools at community levels should be viewed as a vehicle for engagement and learning. We must critically reflect on why we continue to under-invest *despite* our existing knowledge that DRR saves in the longer run, and make adjustments to our strategies accordingly. Second, for such tools to be useful beyond awareness raising, further efforts are certainly needed to systematically develop capacities and to collect locally applicable data in developing countries. Emerging initiatives such as crowd-and-expert-sourced hazard, exposure and vulnerability for example, offers potential avenues to collect risk information in data scarce environment.^24^ Thirdly, it is important to understand that sophisticated quantitative focused tools are not appropriate in all DRR investment appraisals. Depending on scale and scope, less demanding and more qualitative and creative appraisals may be better able to deliver meaningful assessments. Of course, this does not mean we can forget about risk and probability all together – we need a nuanced approach when integrating expert and local knowledge and learning for effective co-production of DRR solutions. Further research and capacity building will certainly be helpful to develop and test alternative evaluation methodologies and building robust empirical evidence in developing countries.

## Competing Interests

The authors have declared that no competing interests exist.
